# Monoaminergic and Opioidergic Modulation of Brainstem Circuits: New Insights Into the Clinical Challenges of Pain Treatment?

**DOI:** 10.3389/fpain.2021.696515

**Published:** 2021-07-05

**Authors:** Isaura Tavares, José Tiago Costa-Pereira, Isabel Martins

**Affiliations:** ^1^Unit of Experimental Biology, Department of Biomedicine, Faculty of Medicine, University of Porto, Porto, Portugal; ^2^Institute of Molecular and Cell Biology, University of Porto, Porto, Portugal; ^3^Institute of Investigation and Innovation in Health, University of Porto, Porto, Portugal; ^4^Faculty of Nutrition and Food Science, University of Porto, Porto, Portugal

**Keywords:** descending pain modulation, opioids, serotonin, noradrenaline, neuropathic pain, dorsal reticular nucleus

## Abstract

The treatment of neuropathic pain remains a clinical challenge. Analgesic drugs and antidepressants are frequently ineffective, and opioids may induce side effects, including hyperalgesia. Recent results on brainstem pain modulatory circuits may explain those clinical challenges. The dual action of noradrenergic (NA) modulation was demonstrated in animal models of neuropathic pain. Besides the well-established antinociception due to spinal effects, the NA system may induce pronociception by directly acting on brainstem pain modulatory circuits, namely, at the locus coeruleus (LC) and medullary dorsal reticular nucleus (DRt). The serotoninergic system also has a dual action depending on the targeted spinal receptor, with an exacerbated activity of the excitatory 5-hydroxytryptamine 3 (5-HT3) receptors in neuropathic pain models. Opioids are involved in the modulation of descending modulatory circuits. During neuropathic pain, the opioidergic modulation of brainstem pain control areas is altered, with the release of enhanced local opioids along with reduced expression and desensitization of μ-opioid receptors (MOR). In the DRt, the installation of neuropathic pain increases the levels of enkephalins (ENKs) and induces desensitization of MOR, which may enhance descending facilitation (DF) from the DRt and impact the efficacy of exogenous opioids. On the whole, the data discussed in this review indicate the high plasticity of brainstem pain control circuits involving monoaminergic and opioidergic control. The data from studies of these neurochemical systems in neuropathic models indicate the importance of designing drugs that target multiple neurochemical systems, namely, maximizing the antinociceptive effects of antidepressants that inhibit the reuptake of serotonin and noradrenaline and preventing desensitization and tolerance of MOR at the brainstem.

## Descending Pain Modulation: General View

The existence of top-down modulation of nociceptive transmission was already postulated by the gate control theory [1]. The periaqueductal gray (PAG) matter, the first brainstem structure with a demonstrated involvement in top-down pain modulation, has reciprocal projections with cortical areas, amygdala [2], and the rostral ventromedial medulla (RVM). The PAG matter does not project directly to the spinal cord (SC) and relays the descending input through the RVM [3], the main serotoninergic spinally projecting neuronal population [4, 5]. Top-down modulation is also mediated by the release of noradrenaline at the dorsal horn of the SC. Descending noradrenergic (NA) projections to the SC arise from three brainstem NA neuronal populations: the A5, A6 (comprising the nucleus subcoeruleus and the LC), and A7 NA cell groups [6–9]. Noradrenaline inhibits nociceptive transmission at the SC through the activation of α_2_-adrenergic receptors (α_2_-AR) located at peripheral nociceptors or spinal neurons [10]. Top-down modulation includes bidirectional control, i.e., inhibitory and facilitatory. Pronociceptive actions are well represented by the medullary dorsal reticular nucleus (DRt), which is reciprocally connected with the dorsal horn of the SC in a reverberating circuit that amplifies nociceptive transmission [11, 12]. Descending facilitation (DF) from the DRt is enhanced in sustained pain models [13, 14] and may account for spinal sensitization during neuropathic pain [15]. The RVM comprises two classes of non-serotoninergic neurons classified by their role in nociceptive modulation: OFF- and ON-cells [16]. The activity of OFF-cells decreases during nociceptive-like behaviors, while the opposite occurs with ON-cells, and OFF-cells were proposed to play antinociceptive effects, whereas ON-cells are likely to exert pronociceptive actions in descending pain modulation [17].

The components of the descending pain modulatory system show neuroplastic changes during neuropathic pain. In the RVM, the imbalance in the activity of ON- and OFF-cells toward the increased activity of the former during traumatic and diabetic neuropathy may facilitate nociceptive spinal transmission [18–20]. The LC also plays bidirectional control of pain modulation. Besides the well-established inhibitory actions through its descending projections to the SC, the LC exerts pain-facilitatory actions through projections to several areas of the pain control modulatory system, such as the DRt [14, 21]. Recent studies described alterations of descending serotoninergic and NA systems and local opioidergic modulation in several neuropathic pain models. These data will be critically analyzed throughout this review to discuss perspectives of designing analgesic drugs that tackle the challenges of neuropathic pain management.

## Descending Monoaminergic Pain Modulation During Neuropathic Pain

**Figure 1:**
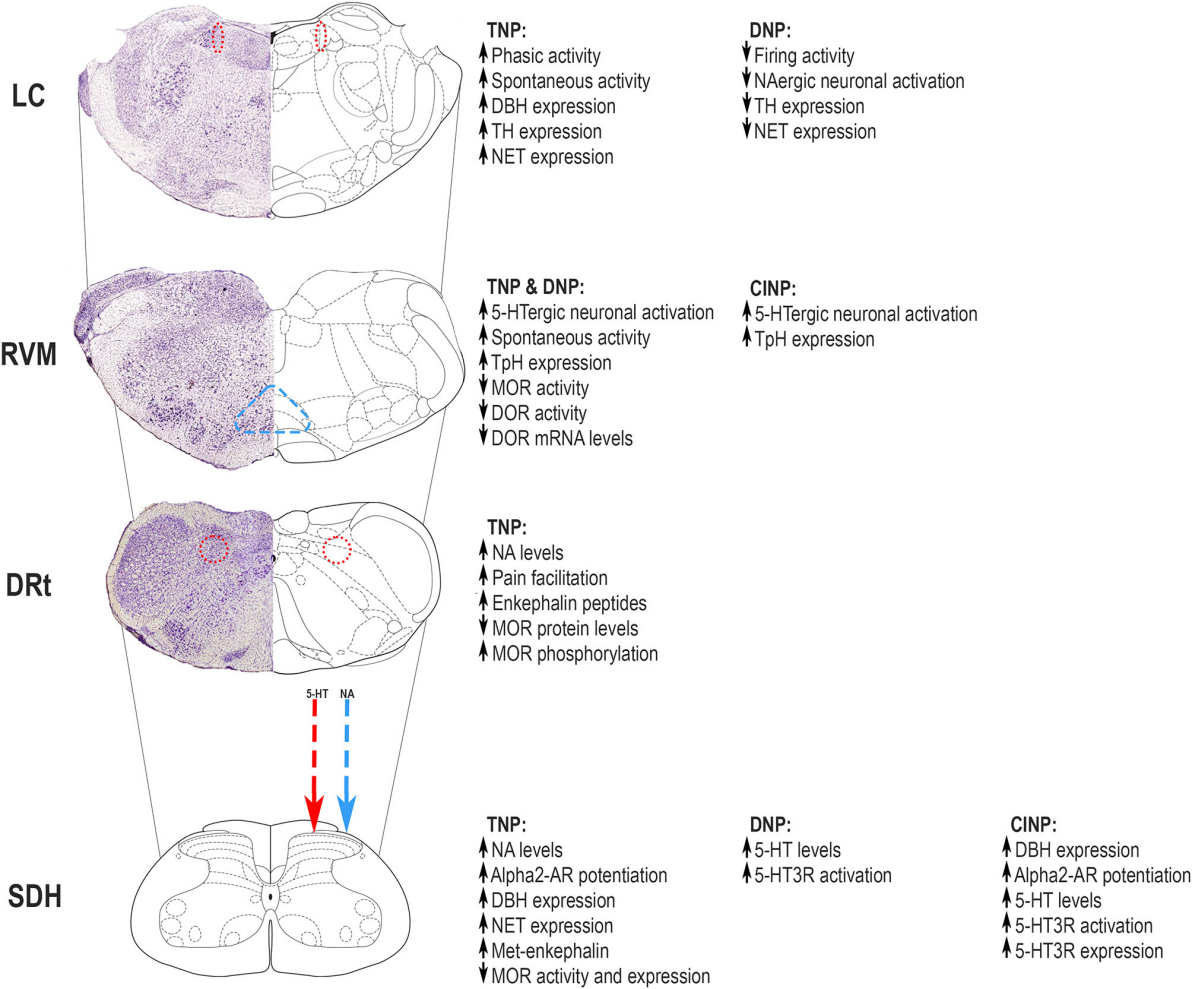
Neuropathic pain induces adaptations in the descending modulatory pain systems involved in serotoninergic and noradrenergic (NA) neurochemical control, some of which are mediated by local opioidergic control. Major changes occur at the locus coeruleus (LC), rostral ventromedial medulla (RVM), and medullary dorsal reticular nucleus (DRt) and affect top-down modulation of nociceptive transmission at the superficial dorsal horn (SDH). The figure summarizes the alterations occurring in different pain models, namely, traumatic neuropathic pain (TNP), diabetic neuropathic pain (DNP), and chemotherapy-induced neuropathic pain (CINP). The mechanisms underlying top-down modulation in different neuropathic pain models are discussed in this review. Adapted from Paxinos and Watson [22]. 5-HT, serotonin; 5-HT3R, 5-HT3 receptor; α_2_-AR - α_2_ adrenoreceptor; DBH, dopamine beta hydroxylase; DOR, delta opioid receptor; DRt, dorsal reticular nucleus; LC, locus coeruleus; MOR, μ-opioid receptor; NA, noradrenergic; NET, noradrenaline transporter; RVM, rostral ventromedial medulla; SDH, superficial dorsal horn; TH, tyrosine hydroxylase; TpH, tryptophan hydroxylase.

The descending serotoninergic and NA systems are altered during neuropathic pain (Figure 1). Regarding the descending serotoninergic modulatory system, an imbalance toward facilitation was detected, which may account for the persistence of pain [23, 24]. In neuropathic pain, descending serotoninergic modulation from the RVM is involved in the maintenance, rather than in the installation, of chronic pain [23, 25]. The depletion of serotoninergic RVM neurons or of serotoninergic pathways reduces nociceptive behaviors after, but not before, nerve injury [26] and prevents nociceptive hypersensitivity in traumatic neuropathic pain (TNP) models [27, 28]. In traumatic and diabetic neuropathic pain (DNP) models, an increase in the serotoninergic input to the SC from hyperactive serotoninergic RVM neurons was proposed to represent an adaptation of descending pain modulation to the increased barrage of nociceptive input [29, 30]. The increased serotonin (5-HT)-mediated input to the SC is likely to be pronociceptive due to the higher activity of facilitatory 5-hydroxytryptamine 3 (5-HT3) receptors at the SC (see below). Further, accounting for pronociceptive actions of the RVM during neuropathic pain, an increase in spontaneous activity of ON-cells was reported in traumatic and DNP models [17]. In a chemotherapy-induced neuropathic pain (CINP) model, similar results were obtained, with higher activation of serotoninergic RVM neurons and increased serotoninergic output to the SC [31]. This indicates that the RVM serotoninergic system is similarly affected at least in some neuropathic pain models. Increases in descending serotoninergic pain recruitment may yield facilitation or inhibition, depending on the targeted subtype of the spinal serotoninergic receptor [32]. The activation of 5-HT1A/B and 5-HT7 spinal receptors inhibits nociception, while the activation of 5-HT3 and 5-HT2A receptors has the opposite effect [32–34]. The administration of 5-HT1A agonists produces strong antinociceptive effects and attenuates depression-like behaviors related to TNP [35], whereas 5-HT1A antagonist reduces or abolishes antinociceptive responses [33, 36, 37]. Spinal 5-HT1A receptors mediate the analgesic effects of cannabidiol during diabetic neuropathy [38]. The systemic administration of 5-HT1A antagonists reduces the antidepressant-like effect of venlafaxine [39]. However, the antidepressant and antinociceptive effects seem to use different groups of 5-HT1A receptors [40]. The administration of the 5-HT7 agonist induces analgesia [41], while the antagonist increases neuropathic pain-like behaviors [42–44]. In contrast, the activation of the 5-HT2A receptors increases pain-like behaviors in models of traumatic and diabetic neuropathy [45, 46]. The 5-HT3R, the only 5-HT ionotropic receptor with excitatory functions, plays a crucial role in pronociception during neuropathic pain [47]. In TNP models, namely, animals with SC injury, intrathecal administration of 5-HT3R antagonist induces antinociception whereas the agonist intensifies allodynia [48, 49]. Moreover, pharmacological spinal blockade of the overexpressed 5-HT3R reverted the neuropathic pain-like behaviors during CINP [31] and attenuated neuronal hyperactivity in diabetic neuropathy [47]. As a whole, the studies indicate that 5-HT3Rs account for central sensitization in neuropathic pain [50]. More studies are necessary to better understand the net balance between the increased serotoninergic input to the SC and the role of spinal receptors, namely, in long-term neuropathic pain models, and also considering the intensity of the noxious stimulus inasmuch that biphasic modulation from the RVM was reported [51].

Neuropathic pain also induces neuroplastic changes in NA pain modulatory areas that may account for pain persistence [52, 53]. Several studies showed that LC neurons present higher electrophysiological responses evoked by noxious stimulation at early stages of nerve injury, but spontaneous activity does not change at that early stages [53–55]. It was shown that, during chronic pain, the balance of LC function may shift from pain inhibition to pain facilitation, which may account for chronic pain installation [56]. A recent study showed higher spontaneous activity and enhanced response of LC neurons after noxious stimulation in long-term traumatic neuropathy [57]. Some reports have also shown that the LC has increased expression of noradrenaline-synthetizing enzymes and noradrenaline transporter (NET) at late stages in long-term traumatic neuropathic pain models [58, 59]. The other NA brainstem cell groups also contribute to the maintenance of neuropathic pain. In the models of diabetic neuropathy, an increase in neuronal activation at the A5 NA cell group was reported at 4 weeks after the induction of diabetes [29], and in traumatic neuropathic models, the administration of α_2_-AR agonist into the A7 NA cell group reduces neuropathic hypersensitivity [60]. The alterations in the NA pain modulatory centers affect nociceptive transmission at the dorsal horn of the SC with a clear involvement of α_2_-AR. In TNP models, NA spinal upregulation occurs with increased spinal noradrenaline levels and enhanced efficacy of G protein-coupled α_2_-AR [61–63]. Furthermore, nerve injury increases the density of NA fibers in the SC, which is associated with increased brain-derived nerve growth factor (BDNF), a neurotrophin involved in neuronal differentiation and neuroplastic pain-related mechanisms [64]. Moreover, upregulation of the spinal NET was reported in TNP models [65]. With the progression of traumatic neuropathy, a gradual loss of descending NA inhibition occurs [66]. In the CINP animal model, increased expression of NA biosynthetic enzymes at the dorsal horn and the potentiation of the α_2_-AR-mediated antinociception at the SC were recently described [67]. Although the studies of NA descending modulation have been mostly directed to α_2_-AR, recent demonstrations that noxious stimulation activates astrocytes at the superficial dorsal horn (SDH) through α_1_A-AR [68] will open new avenues for pain research in the future.

Besides the direct effects in the direct input to the SC, the alterations of the serotoninergic and NA systems also affect the brainstem pain modulatory system. The LC and A5 NA cell groups project to the DRt [12]. The increased activation of NA LC and A5 neurons in traumatic neuropathic models leads to increased release of noradrenaline into DRt, which was proposed to enhance DF of nociceptive transmission from that medullary area [14]. The studies of the endogenous pain modulatory system should, therefore, be performed considering the connectivity between areas, which is a clinically relevant issue. Patients with neuropathic pain have higher complaints of pain when the PAG-RVM connectivity is stronger [69]. The enhanced recruitment of descending NA inhibition during CINP likely aims to compensate for the increased 5-HT3-mediated descending serotoninergic facilitation from the RVM [67]. Preclinical studies would benefit from shifting from studying a single pain modulatory system in the brain to studying about approaching the connectivity issues, namely, in what concerns the interplay between the serotoninergic and NA systems.

## Opioidergic Modulation of Brainstem Pain Control Circuits

Endogenous opioids are involved in the control of the descending pain modulatory system through the activation of mu (MOR), delta (DOR), kappa (KOR), and nociceptin opioid peptide (NOP) receptors [70]. The endogenous opioids, such as enkephalins (ENKs), β-endorphins, and dynorphins, bind, by order of preference, to DOR, MOR, and KOR, respectively. Regarding nociceptin, it binds to NOP and its role as an independent neural “anti-opioid” system has been proposed [71].

**Figure 2:**
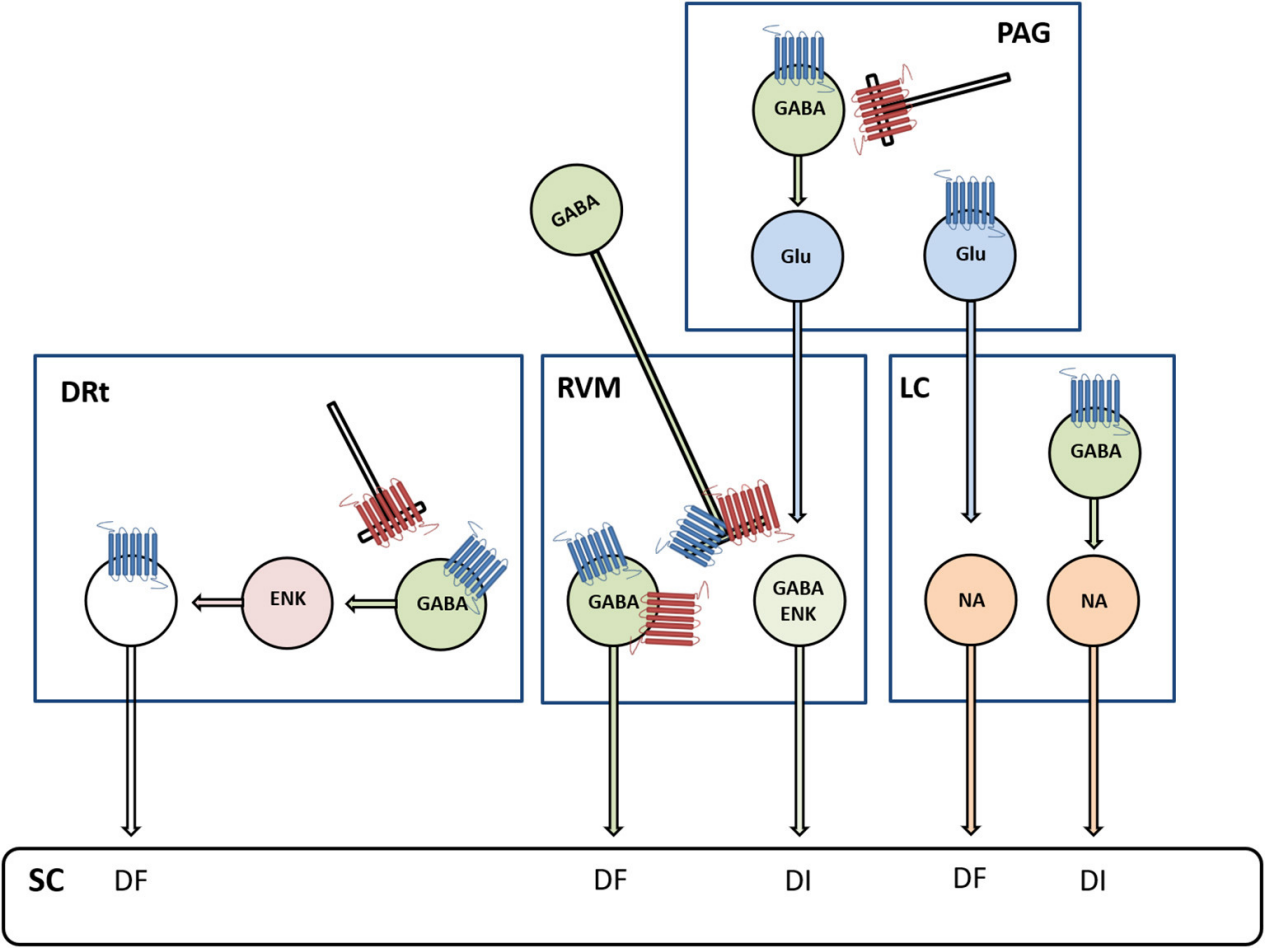
Diagram depicting the involvement of μ-opioid receptors (MOR, marked in blue) and δ-opioid receptors (DOR, marked in red) in descending modulation from the periaqueductal gray (PAG) matter, RVM, LC, and the DRt. Opioids are involved in the mediation of descending inhibition (DI) and descending facilitation (DF) from PAG circuits relayed in the RVM and LC, respectively. In the PAG-RVM circuit, MOR- and DOR-mediated inhibition of gamma aminobutyric acid (GABA)ergic neurons disinhibits glutamatergic (Glu) neurons projecting to the RVM. In the PAG-LC circuit, MOR inhibits a subtype of Glu neurons, which express the phospholipase C β4, projecting to NA LC neurons. This newly discovered circuit is thought to counterbalance the adverse excitatory effects of stress on the LC-NA system. Opioids are also involved in the mediation of DI from the LC through MOR-mediated inhibition of GABAergic neurons that disinhibit NA neurons projecting to the spinal cord (SC). Opioids in the RVM produce antinociception *via* direct inhibition of pronociceptive cells expressing MOR and DOR, which are GABAergic and functionally correspond to ON-cells, and indirect activation (i.e., disinhibition) of antinociceptive cells, which co-express GABA and enkephalins (ENKs) and functionally correspond to OFF-cells. Opioids in the DRt act through direct inhibition of DRt spinally projecting neurons, which express MOR, and indirectly through activation (i.e., disinhibition) of local ENK neurons.

The PAG and RVM constitute major sites of supraspinal MOR analgesia [72, 73]. Genetic approaches confirmed that MOR activates the PAG-RVM descending pathway *via* suppression of the inhibitory influence of local GABAergic interneurons (Figure 2) [74, 75]. The administration of opioids in the RVM produces antinociception through direct inhibition of pronociceptive MOR-expressing ON-cells and indirect activation (i.e., disinhibition) of antinociceptive OFF-cells [17]. The neurochemical nature and synaptic mechanisms of the PAG-RVM circuitry were recently addressed using genetic approaches [76]. Neurons co-expressing gamma aminobutyric acid (GABA) and preproenkephalin functionally correspond to OFF-cells and directly project onto nociceptor terminals in the dorsal horn to inhibit nociceptive transmission (Figure 2) [77]. Other GABAergic RVM neurons express MOR and project to preproenkephalin dorsal horn interneurons, facilitating the transmission of nociceptive information [76]. The activation of DOR and KOR also modulates the PAG-RVM circuit. The mechanisms and consequences of DOR activation in the PAG-RVM circuit are similar to MOR (Figure 2) [78, 79]. DOR agonists typically show lower adverse effects than MOR agonists, but their efficacy is also lower, probably due to intracellular trafficking [80]. The administration of KOR agonists in the RVM inhibits OFF-cells and blocks the antinociceptive actions of MOR activation [81, 82]. The NOP receptor is abundant in the PAG and RVM [83]. The role of the nociceptin/orphanin FQ peptide (N/OFQ)–NOP receptor system is better studied in the RVM, and NOP is expressed in OFF-cells and co-expressed with MOR in ON-cells (Figure 2) [84, 85]. Supraspinal N/OFQ induces pronociceptive effects along with an anti-opioid analgesic action [86]. In contrast, N/OFQ attenuates opioid withdrawal-induced hyperalgesia by inhibiting ON-cells [84]. Given the role of activation of ON-cells in the maintenance of neuropathic pain [87], the inhibition of ON-cells likely contributes to the antihyperalgesic and antiallodynic effects of systemic and supraspinal NOP ligands in neuropathic pain models [88, 89].

The effects of opioids have also been extensively studied in the LC, where MOR is highly expressed [90]. Opioid receptors in the LC are implicated in pain modulation, stress responses, and opioid drug effects [91, 92]. Early studies indicate that opioids produce antinociception by enhancing the descending NA inhibition [93]. In the extreme, opioids inhibit LC neurons, and following chronic morphine infusion, LC neurons undergo desensitization, accounting for tolerance to opioids [94]. Opioidergic modulation of the LC is complex as opioids can also suppress descending inhibition (DI) through the PAG-LC pathway (Figure 2) [74]. In this respect, the opioidergic system has two different roles at the PAG: one enhances DI, through the PAG-RVM pathway, and the other suppresses DI, through the PAG-LC pathway. Notwithstanding, the final output to spinal nociceptive information will likely further involve the opioidergic modulation of upstream brain structures, such as the amygdala, with which the PAG is connected [95].

The LC plays a complex role in pain modulation with facilitatory and inhibitory modes of modulation of nociception. It exerts pain-facilitatory actions through its ascending projections to several supraspinal areas [13, 14, 21] and inhibitory actions through its descending projections to the SC. Opioids produce antinociception partly by enhancing the descending NA inhibition [96–99]. LC neurons have also been implicated in tolerance to opioids. The LC has a high density of MORs [90, 100]. In the extreme, opioids inhibit LC neurons; however, following chronic treatment with morphine, LC neurons undergo desensitization, which contributes to the development of tolerance to opioid effects (reviewed by 91). The desensitization of LC neurons was inhibited in mice expressing a mutant MOR that renders the receptor increasingly unable to interact with β-arrestins, and these exhibit enhanced opioid-induced analgesia [101].

At the DRt, opioids are a key local modulatory system that can directly and indirectly modulate the spinal-DRt-spinal reverberative pathway. Opioids act through direct inhibition of DRt spinally projecting neurons that express MOR and through disinhibition of enkephalinergic interneurons that receive input from GABAergic interneurons that express MOR (Figure 2) [102, 103]. These GABAergic interneurons are also presynaptically inhibited by DOR-expressing fibers [102]. Local overexpression of opioid peptides, namely ENK, was shown to inhibit DRt pain facilitation [104]. The activation of MOR at the DRt plays a fundamental inhibitory role at the DRt and was recently shown to account for the analgesic effects of systemic opioids [105].

The supraspinal opioidergic modulation may account for neuropathic pain. During neuropathic pain, the increased DF is not compensated by enhanced DI [50, 87]. The engagement of DI mediated by the RVM-OFF cells, through which opioids produce descending pain inhibition, protects against the development of neuropathic pain [106]. Evidence of a dysfunctional descending opioidergic inhibition in neuropathic pain is further provided by the decrease of diffuse noxious inhibitory control (DNIC) in animal models [107–111]. DNIC is mainly a neurophysiological phenomenon, and many authors consider [102–104] that its psychophysical paradigm in humans is represented by conditioned pain modulation (CPM). DNIC is a unique form of endogenous analgesia that requires descending inhibitory pathways [73] and is partly mediated by opioids [107, 112]. Tapentadol, a MOR agonist and noradrenaline reuptake inhibitor [113], can restore DNIC/CPM [108, 114]. The effects of tapentadol are mostly attributed to a synergistic effect of MOR activation and the inhibition of noradrenaline reuptake at the SC [115]. In the SC, MOR serves as an interface for ascending inhibition and descending opioidergic inhibition triggered from PAG-RVM [70, 116].

During neuropathic pain, the endogenous opioid peptides and opioid receptors are altered at the supraspinal pain control system. Local release of endogenous opioids was shown in cortical and subcortical brain areas of patients with persistent neuropathic pain [117–120]. In animal models of neuropathic pain, increased ENK peptide levels were detected in several components of the supraspinal pain control system, including the PAG, RVM, and DRt [121, 122]. The role of endogenous opioid peptides has been studied using knockout mice deficient in opioid-encoding genes revealing deficits in supraspinal modulation [123]. Dynorphin knockouts showed the involvement of dynorphins in the affective component of pain. Increased release of dynorphin together with increased KOR signaling was recently detected in the mesolimbic circuit and amygdala, and the upregulation of this system was responsible for mediating the aversiveness/unpleasantness of neuropathic pain [124, 125]. The role of dynorphin in the amygdalo-parabrachial pathway and its involvement in emotional and pain control were recently proposed [126]. The studies performed in β-endorphin knockout mice suggest that the continuous release of β-endorphin induces activation of MOR and subsequent phosphorylation and desensitization [127–129]. Mice lacking proenkephalin and/or β-endorphin showed that these peptides modulate the activity and the levels of MOR, DOR, and KOR in descending pain control areas of the brainstem [130]. Neuropathic pain is associated with a reduction in MOR function in the brainstem, with decreased activation of G proteins likely due to increased phosphorylation of the receptor, leading to its desensitization [131]. Reduced MOR-mediated G-protein activity was shown in the PAG [132] and RVM [133] of neuropathic pain models. At the DRt, we recently showed that neuropathic pain leads to increased release of ENK peptides and desensitization of MOR [105]. Additionally, we showed a reduction in protein levels of MOR and an increase in phosphorylation of MOR [105]. The reduction in MOR protein was likely associated with increased phosphorylation, leading to desensitization and subsequent degradation of the receptor, since no alteration in mRNA levels of MOR was detected [105]. These molecular adaptations of MOR impair the analgesic function of MOR at the DRt [105]. Neuropathic pain is associated with altered expression of the opioid receptors [105, 133] at several supraspinal pain modulatory areas. Downregulation of MOR in the brain seems to be common during neuropathic pain [134, 135]. In patients with neuropathic pain, reduced MOR availability was observed in cortical brain areas involved in pain modulation and in the PAG [119, 120, 136]. DOR was also found to be downregulated in the RVM after nerve injury [133]. Recent characterization of DOR confirmed its relevance in the development of neuropathic pain. DOR knockouts developed increased thermal and mechanical sensitivities in neuropathic pain models [137], suggesting a protective role of DOR. At the RVM, the downregulation of DOR together with the desensitization of MOR [133] likely contributes to a decrease in descending opioidergic inhibition. The downregulation of MOR at SC and descending pain modulatory areas likely contributes to the reduced potency of morphine in the neuropathic pain state [105, 138, 139].

## Clinical Implications of the Alterations in Descending Modulatory Systems During Neuropathic Pain

The treatment of neuropathic pain remains a challenge since many drugs show inadequate analgesia and considerable side effects. The studies of brainstem pain modulation in preclinical models of neuropathic pain unraveled some mechanisms that may account for the inadequate analgesia and indicate possible approaches to overpass the challenges of neuropathic pain management. Drugs with a primary function other than analgesia are used for neuropathic pain treatment, such as antidepressants that act upon the reuptake of serotonin and noradrenaline [140]. Antidepressants that act at the serotoninergic system have their net analgesic efficacy reduced by the increased activity of spinal 5-HT receptors involved in pain facilitation, namely, 5-HT3 [32, 44–46]. Regarding antidepressants that also inhibit noradrenaline reuptake, such as duloxetine [141], the analgesic effects due to inhibition of α_2_-AR at the SC may be attenuated by the pronociceptive effects of noradrenaline at supraspinal pain control centers [13, 14].

Weak and strong opioids are recommended as second- and third-line treatments, respectively, mainly because of lack of efficacy and safety concerns [reviewed by [140, 142, 143]]. The lack of effectiveness of MOR-acting drugs in neuropathic pain might be because both neuropathic pain and opioid treatment lead to desensitization of and tolerance to MOR [144]. Tolerance to opioids leads to increasing doses of opioids, which is critical as this can lead to opioid-induced hyperalgesia [145]. Chronic morphine treatment induces a shift of MOR signaling from inhibitory to excitatory at the DRt, enhancing DF from the DRt [146]. These findings indicate that the cumulative effects of neuropathic pain and opioid drugs on MOR are counterproductive. In the subsequent years, the study of the brainstem pain control system needs to have a more translational perspective. The effects of descending modulation should also consider interactions occurring at both local neurochemical modulation of supraspinal pain control circuits and between pain control centers of the brain. Imaging studies in human subjects have recently shown changes in the functional connectivity of pain control centers of the brain [147], and this “connectome perspective” should also be considered in the basic pain research studies. The involvement of opioids in the control of the connections and as a trigger of opioid-induced hyperalgesia should be considered. These perspectives may allow to overcome the current gaps in research studies deriving from the moderate translation of basic studies of brainstem pain modulatory circuits.

## Author Contributions

IT and IM contributed to the initial conception of the paper. JC-P wrote the first draft of the 2 initial sections of the manuscript. IM and IT wrote the third and fourth sections, respectively. All authors contributed to manuscript revision and approved the submitted version.

## Conflict of Interest

The authors declare that the research was conducted in the absence of any commercial or financial relationships that could be construed as a potential conflict of interest.
